# Oridonin Attenuates Thioacetamide-Induced Osteoclastogenesis Through MAPK/NF-κB Pathway and Thioacetamide-Inhibited Osteoblastogenesis Through BMP-2/RUNX2 Pathway

**DOI:** 10.1007/s00223-023-01080-5

**Published:** 2023-04-09

**Authors:** XiaoLi Jin, Jia Xu, Fanfan Yang, Jin Chen, Feng Luo, Bin Xu, Jian Xu

**Affiliations:** 1 School of Medical Technology and Information Engineering, Zhejiang Chinese Medical University, Hangzhou, Zhejiang 310053 People’s Republic of China; 2 Department of Clinical Laboratory, The First Affiliated Hospital of Zhejiang Chinese Medical University, Hangzhou, 310006 China; 3 Department of General Surgery, School of Medicine, Sir Run Run Shaw Hospital, Zhejiang University, Hangzhou, Zhejiang 310016 People’s Republic of China

**Keywords:** Thioacetamide, Oridonin, Osteoclastogenesis, Osteogenesis, Therapeutics

## Abstract

Osteoporosis, an age-related metabolic bone disease, is mainly caused by an imbalance between osteoblast-mediated bone formation and osteoclast-mediated bone resorption. At present, there are many osteoporosis drugs that can promote bone formation or inhibit bone resorption. However, there were few therapeutic drugs that can simultaneously promote bone formation and inhibit bone resorption. Oridonin (ORI), a tetracyclic diterpenoid compound isolated from Rabdosia rubescens, has been proved to have anti-inflammatory, anti-tumor effects. However, little is known about the osteoprotective effect of oridonin. Thioacetamide (TAA) is a common organic compound with significant hepatotoxicity. Recent studies have found that there was a certain association between TAA and bone injury. In this work, we investigated the effect and mechanism of ORI on TAA-induced osteoclastogenesis and inhibition of osteoblast differentiation. The results showed that TAA could promote the osteoclastogenesis of RAW264.7 by promoting the MAPK/NF-κB pathway, and also promoted p65 nuclear translocation and activated intracellular ROS generation, and ORI can inhibit these effects to inhibit TAA-induced osteoclastogenesis. Moreover, ORI can also promote the osteogenic differentiation pathway and inhibit adipogenic differentiation of BMSCs to promote bone formation. In conclusion, our results revealed that ORI, as a potential therapeutic drug for osteoporosis, could protect against TAA-induced bone loss and TAA-inhibited bone formation.

## Introduction

Osteoporosis is one of the major epidemics of the twenty-first century, affecting about 200 million people around the world [1], which seriously endangers people's health. As a dynamic organ, bone mass maintenance depends on the balance between osteoblast-mediated bone formation and osteoclast-mediated bone resorption [2]. The imbalance between the activity of osteoblasts and osteoclasts will lead to increased bone fragility and increased incidence of fractures [3]. Although estrogen, calcitonin, parathyroid hormone, and bisphosphonates have been widely used to against osteoporosis, there are still limitations exist in the treatment of osteoporosis [4]. Based on the above issues, new treatment methods for osteoporosis should include suppressing bone resorption and potentiating bone formation.

In recent years, natural drug monomers extracted from Chinese herbal medicines are getting increasingly attention because of their antioxidant [5, 6], anti-inflammatory [7, 8], and anti-cancer effects [9], and have become potential targets for the treatment of various diseases. Oridonin (ORI), a tetracyclic diterpenoid compound extracted from Rubescens, has a variety of therapeutic effects and has been widely used in research, especially in anti-inflammatory [10] and anti-cancer effects [11]. It was found that ORI could inhibit the proliferation and migration of bladder cancer cells (human T24 BC cells) by blocking the phosphorylation of ERK and AKT and down-regulating the expression of TRPM24 [12]. ORI can also inhibit the inflammatory reaction of diabetes nephropathy rats, reduce the level of inflammatory cytokines, and alleviate the infiltration of inflammatory cells in renal tissue through inhibiting TLR4/p38-MAPK and TLR4/NF-κB pathways [13]. As a specific covalent inhibitor of NLRP3 inflammasome, ORI could specifically inhibit the activation of NLRP3 inflammasome. Jiang found that ORI can promote osteogenic differentiation of LPS-induced human periodontal ligament stem cells (hPDLSCs) through inhibiting the NF-κB/NLRP3 pathway [14]. Recent studies have found that ORI can inhibit the proliferation of osteoclast precursor cells and suppress the osteoclast differentiation, suggesting that ORI has a potential anti-bone resorption effect. More specifically, ORI can prevent ovariectomy-induced bone loss in vivo, and regulate Ifrd1-mediated and IκBα-mediated nuclear translocation of p65 to inhibit osteoclastogenesis in vitro [15]. In addition, ORI can regulate the MAPK pathway involved in osteoclast differentiation by inducing phosphorylation of ERK, JNK and p38 [16].

Thioacetamide (TAA), a classic compound that establishes liver fibrosis in animals, mainly induces oxidative stress and inflammation [17]. As early as 1984, Lassila V [18, 19] has proposed that TAA-induced liver injury was accompanied by a reduction in alveolar bone and new bone formation. Interestingly, Nakano [20] also found decreased bone volume and increased bone resorption in TAA-induced cirrhotic rats. Our previous research found that intraperitoneal injection of TAA can induce cortical bone damage and increase trabecular separation in SD rats [21–23]. In addition, TAA, which has the same effect as the classical osteoclast inducers RANKL and M-CSF, can induce BMMs differentiate into osteoclasts in vitro by inducing phosphorylation of PI3K, AKT, p38 and JNK and upregulating the expression of osteoclast specific proteins TRAP and cathepsin K [23]. TAA can also enhance the activity of osteoclasts and promote bone resorption [21]. However, the effect of TAA on osteogenic differentiation is still unclear. Osteoblasts can be differentiated from bone mesenchymal stem cells (BMSCs). In addition to osteoblasts, BMSCs can also be able to differentiate into adipocytes, resulting in a relative reduction of osteoblasts [24]. Many factors are involved in regulating the osteogenic and lipogenic transformation of BMSCs, among which the Wnt/β-catenin pathway is crucial. The expression of key osteogenic transcription factors RUNX2 and β-catenin can promote the osteogenic transformation of BMSCs, while the key lipogenic transcription factor PPARγ can regulate the adipogenic differentiation of BMSCs [25].

At present, there are no studies to demonstrate the effect of ORI in the treatment of TAA-induced bone injury. Therefore, we aimed to investigate the effect of ORI, a potential anti-bone resorption and bone-promoting drug, on TAA-induced osteogenic and osteoclast differentiation and specific molecular mechanism.

## Methods

### Cells and Cell Culture

RAW264.7 cells were purchased from National Collection of Authenticated Cell Cultures. Shanghai SLAC Laboratory Animal Co, Ltd provided the Sprague–Dawley (SD) rats. BMSCs were isolated from SD rats born 1–10 days as previously described [26]. Both RAW264.7 and BMSCs were cultured in DMEM supplemented with 1% penicillin/streptomycin and 10% FBS (fetal bovine serum; Sigma). The cells were cultured in a humidified environment at 37 °C with 5% CO_2_.

### Preparation of TAA

ORI was purchased from Med Chem Express, and TAA was purchased from Sang on Biotech (> 98.0% purity; China). RANKL and M-CSF were obtained from PEPRO TECH. The p38 MAPK inhibitor (SB202190) and NF-κB inhibitor (PPM-18) were supplied by APExBIO.

### Cell Cytotoxicity Assay (CCK-8)

The cytotoxicity of TAA and ORI on RAW264.7 and BMSCs was measured by CCK-8. More specifically, cells were planted into 96-well plates (5 × 10^3^ cells/well) and cultured with different concentrations of TAA (0, 0.5, 1, 1.5, and 2 mg/mL) or ORI (0, 0.84, 1.69, 3.38, 6.75 and 13.5 μM) for different times. Then, DMEM supplemented with CCK-8 solution was added to each well for continuous culture for 2 h. Spectrophotometer was used to detect the absorbance at 450 nm.

### Tartrate-Resistant Acid Phosphatase (TRAP) Staining

RAW264.7 were seeded in a 6-well plate at a density of 1** × **10^6^ cells/well (control: cells cultured with DMEM; RANKL + M-CSF: 50 ng/mL RANKL and 30 ng/mL M-CSF; TAA: 1 mg/mL TAA; TAA + ORI: 1 mg/mL TAA and 3.38 μM ORI) to induce osteoclast formation in vitro. TRAP staining was used to identify osteoclasts. After 7 days, osteoclastogenesis was identified by TRAP staining. TRAP^+^ cells were considered to contain three or more nuclei.

### Immunofluorescence

RAW264.7 were plated in 6-well plate (1** × **10^6^ cells/well) and cultured with RANKL + M-CSF or TAA or TAA + ORI for 7 days. Then the cells were fixed in 4% paraformaldehyde for 30 min and permeated with 0.2% Triton X-100 for 15 min. Then block with 5% BSA for 1 h and incubate with primary antibody (anti-P65, 1:100; anti-TRAP, 1:100) or F-actin (1:200) at room temperature for 1 h. PBS was used to wash away the unbound antibody and incubated the secondary antibody (goat anti-rabbit IgG, 1:200) at room temperature for 1 h. The cells were washed with PBS and stained with 4’,6-diamidino-2-phenylindole (DAPI) for 5 min. The cells were observed and photographed by fluorescence confocal microscope.

### Western Blot

Western blot was used to detect the expression of different proteins in RAW264.7 and BMSCs. Anti-c-Fos (Beyotime, 1:2000), anti-NFATc1(ABclonal, 1:3000), anti-TRAP(Abcam, 1:5000), anti-cathepsin-K(Abcam, 1:2000), anti-TLR4(Sangon Biotech, 1:2000), anti-p-p65(Beyotime, 1:2000), anti-p65(ABclonal, 1:2000), anti-p-IκBα(ImmunoWay, 1:2000), anti-IκBα(ImmunoWay, 1:2000), anti-p-ERK (ABclonal, 1:2000), anti-ERK(ABclonal, 1:2000), anti-p-p38(ABclonal, 1:2000), anti-p38 (ABclonal, 1:2000), anti-p-JNK(ABclonal, 1:2000), anti-JNK(Abcam, 1:2000), anti-BMP-2(Abcam, 1:2000), anti-RUNX2(Abcam, 1:2000), anti-β-catenin(Abcam, 1:10,000), anti-p53(Abcam, 1:2000), anti-bax (Abcam, 1:10,000), anti-bcl-2(Abcam, 1:2000), anti-PPAR-γ(Solarbio, 1:2000), anti-β-actin(Abcam, 1:2000), goat anti-mouse IgG(Abcam, 1:5000), goat anti-rabbit IgG(Abcam, 1:5000) were used for protein analysis. ECL substrate kit (BL520B, biosharp) was used for visual analysis of proteins. The gray values of the bands were quantified by ImageJ software.

### Reactive Oxygen Species (ROS) Determination

BMMs were cultured with RANKL + M-CSF or TAA or TAA + ORI for 2 h, then cells were incubated in the dark at 37° C in serum-free medium containing 10 mM DCFH-DA probes (Beyotime, S0033S) for 20 min. The cells were washed three times in serum-free medium to remove extracellular probes. Cells were immediately examined by flow cytometry**.**

### Alkaline Phosphatase (ALP) Staining and Alizarin Red Staining

BMSCs were cultured in osteogenic induction medium (50 mg/ml of ascorbic acid, 0.04 mg/ml dexamethasone and 10 mM β-glycerol-phosphate) containing TAA, ORI or TAA + ORI for 14 days, and wash three times with PBS. The cells were stained with ALP stain kit (code No.294–67,001) and alizarin red kit (Solarbio, G8550), and observed and photographed under optical microscope.

### Apoptosis Assays

BMSCs were seeded at the density of 10^6^ cells per plates, and treated with osteogenic induction medium containing TAA, ORI or TAA + ORI for 14 days. Then the cells were collected and stained with Annexin V-FITC/PI apoptosis kit (Multi sciences, AP101). Flow cytometry was used to detect apoptosis.

### Assay of Adipogenic Differentiation

BMSCs were cultured in adipogenesis induction medium (5 μg/mL insulin, 0.5 mmol/L 3‐isobutyl‐1‐methylxanthine, and 1 μmol/L dexamethasone) containing TAA, ORI or TAA + ORI for 14 days. BODIPY staining (GLPBIO, GC42959-5, 1:1000 dilution) and oil red O staining (Solarbio, G1262) were used to detect the formation of adipocytes.

### Statistical Analysis

Statistical analysis was performed using Statistical Program for Social Sciences (SPSS) software 19.0. Statistical analysis of all data was performed using GraphPad Prism 7(GraphPad Software). *P* < 0.05 was considered statistically significant. Each experiment was repeated at least three times in vitro. The results from at least three independent experiments were presented as the mean ± SD.

## Results

### TAA and ORI Affect RAW264.7 Proliferation

**Fig. 1:**
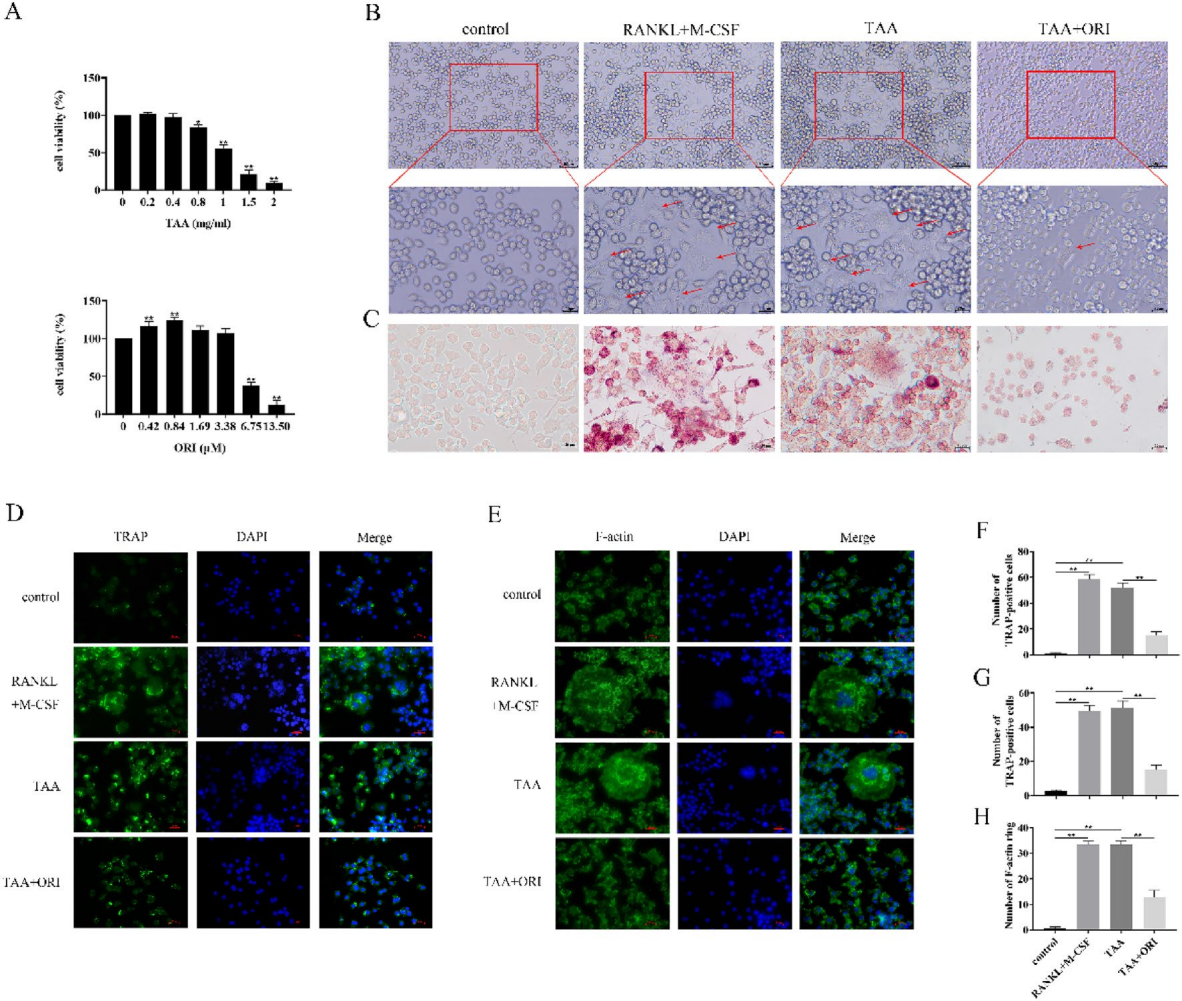
TAA induced osteoclast differentiation of RAW264.7, but ORI could inhibit TAA-induced osteoclast differentiation. **A** RAW264.7 were treated with TAA (0, 0.2, 0.4, 0.8, 1, 1.5, 2 mg/mL) or ORI (0,0.42, 0.84, 1.69, 3.38, 6.75, 13.5 μM) for 48 h, then cell viability was measured by CCK-8. **B** RAW264.7 was cultured with different medium (50 ng/mL RANKL and 30 ng/mL M-CSF; 0.4 mg/ml TAA; 0.4 mg/ml TAA and 3.38 μM ORI) for 7 days, and the cell morphology was observed under the microscope. **C–D** Osteoclastogenesis was identified by TRAP staining. **E **The formation of F-actin was observed by immunofluorescence staining. **F–G** TRAP-positive multinucleated cells (nuclei > 3) were counted (F for TRAP staining, G for Immunofluorescence). **H** Quantification of the number of F-actin rings. Values were expressed as mean ± SD of three individual experiments; *P < 0.05, **P < 0.01

In order to explore the molecular mechanism of the effect of TAA and ORI on osteoclast differentiation, CCK-8 assay was used to analyze the potential cytotoxicity on RAW264.7. As shown in Fig. 1A, TAA had toxic effects on RAW264.7 in a dose-dependent manner. In contrast, low-dose(0.42, 0.84, 1.69, 3.38 μM) ORI can promote the proliferation of RAW264.7, while high-dose(6.75, 13.5 μM) ORI significantly inhibits cells proliferation. Therefore, we selected 0.4 mg/ml TAA and 3.38 μM ORI, which did not affect cell proliferation, as subsequent experimental concentrations to study the effects of TAA and ORI on osteoclast differentiation.

### ORI Attenuates TAA-Induced Osteoclast Differentiation

To determine the effect of TAA and ORI-treated RAW264.7 osteoclast differentiation, TRAP and F-actin staining were observed. Exposed to TAA, RAW264.7 cells became large and irregular, with a lot of purplish granules in the cytoplasm and an increased number of nucleuses (Fig. 1B, C). TRAP immunofluorescence staining also showed increased TRAP expression in TAA group (Fig. 1D). In general, there was no significant difference in the number of TRAP-positive cells and the number of F-actin rings between RANKL + M-CSF treatment or TAA treatment during the induction of osteoclast differentiation (Fig. 1C–E, both were significantly higher than those in the control group). This indicates that TAA can induce osteoclastogenesis independently of RANKL + M-CSF. However, under ORI treatment, the number of TRAP-positive cells (Fig. 1F, 71.34% decrease vs TAA group; Fig. 1G, 70.78% decrease vs TAA group) and the number of F-actin rings (Fig. 1H, decrease 61% vs TAA group) were significantly reduced. More specifically, ORI can inhibit the expression of TRAP and the formation of F-actin.

### ORI Attenuates TAA-Induced Osteoclast-Specific Gene Expression

**Fig. 2:**
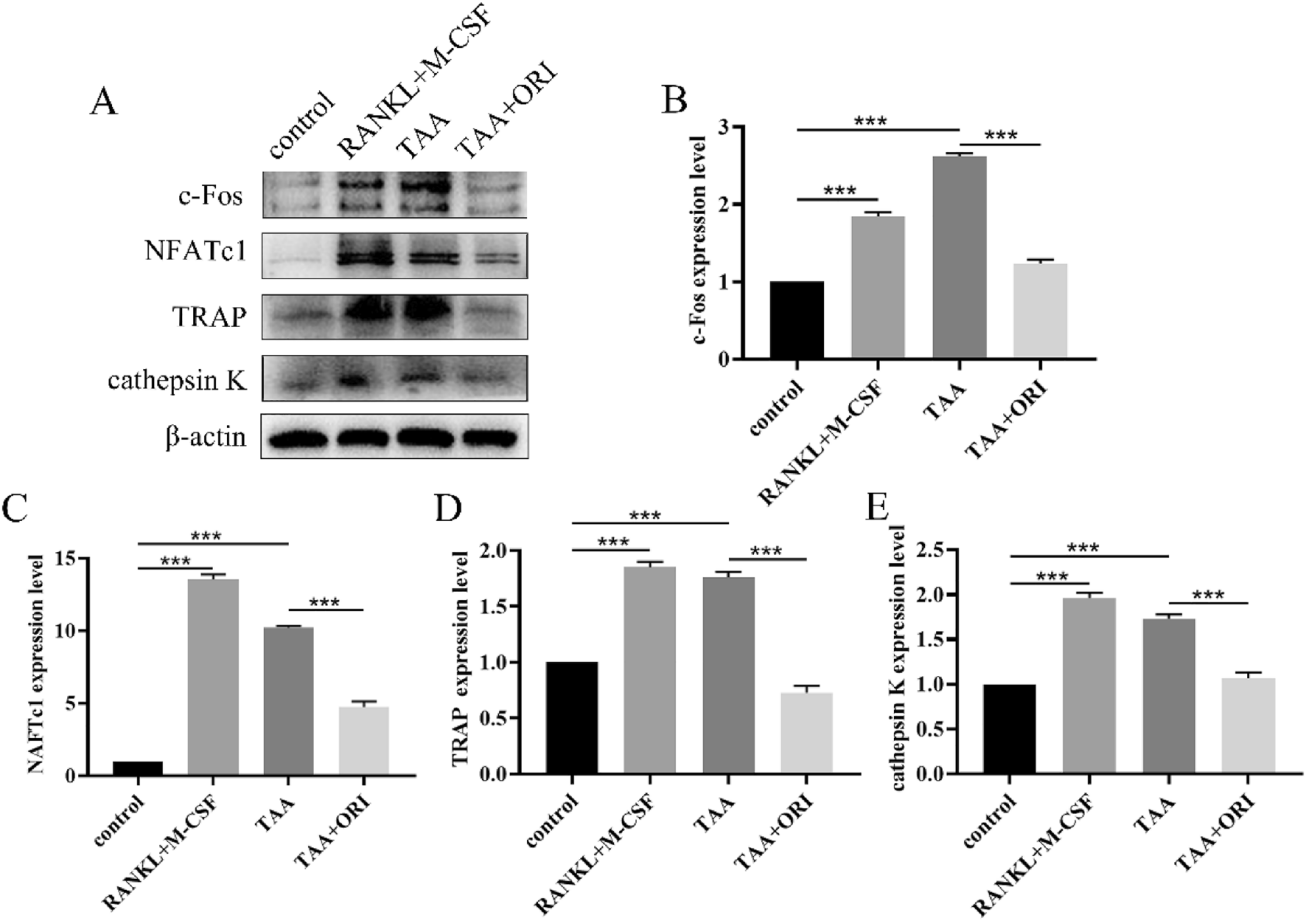
ORI inhibits TAA-induced osteoclast-specific gene expression. **A** Cells were cultured with different medium (50 ng/mL RANKL and 30 ng/mL M-CSF; 0.4 mg/ml TAA; 0.4 mg/ml TAA and 3.38 μM ORI) for 7 days, and Western blot was used to detect the expression of osteoclast-specific proteins of c-Fos, NFATc1, TRAP and cathepsin K. **B–E** The band intensity ratio of c-Fos, NFATc1, TRAP and cathepsin K relative to β-actin were quantified. Values were expressed as mean ± SD of three individual experiments; ***P < 0.001

Osteoclast differentiation is often accompanied by increased expression of osteoclast-specific genes. In order to explore the specific molecular basis of TAA and ORI on osteoclast differentiation, western blot was used to detect the expression of osteoclast-specific gene, including c-Fos, NFATc1, TRAP and Cathepsin K. The results showed that TAA, which has the same effect as RANKL, can up-regulate the expression of osteoclast-specific gene, while ORI can inhibit their expression (Fig. 2).

### ORI Suppresses TAA-Induced Osteoclastogenesis by Inhibiting MAPK/NF-κB Pathways

**Fig. 3:**
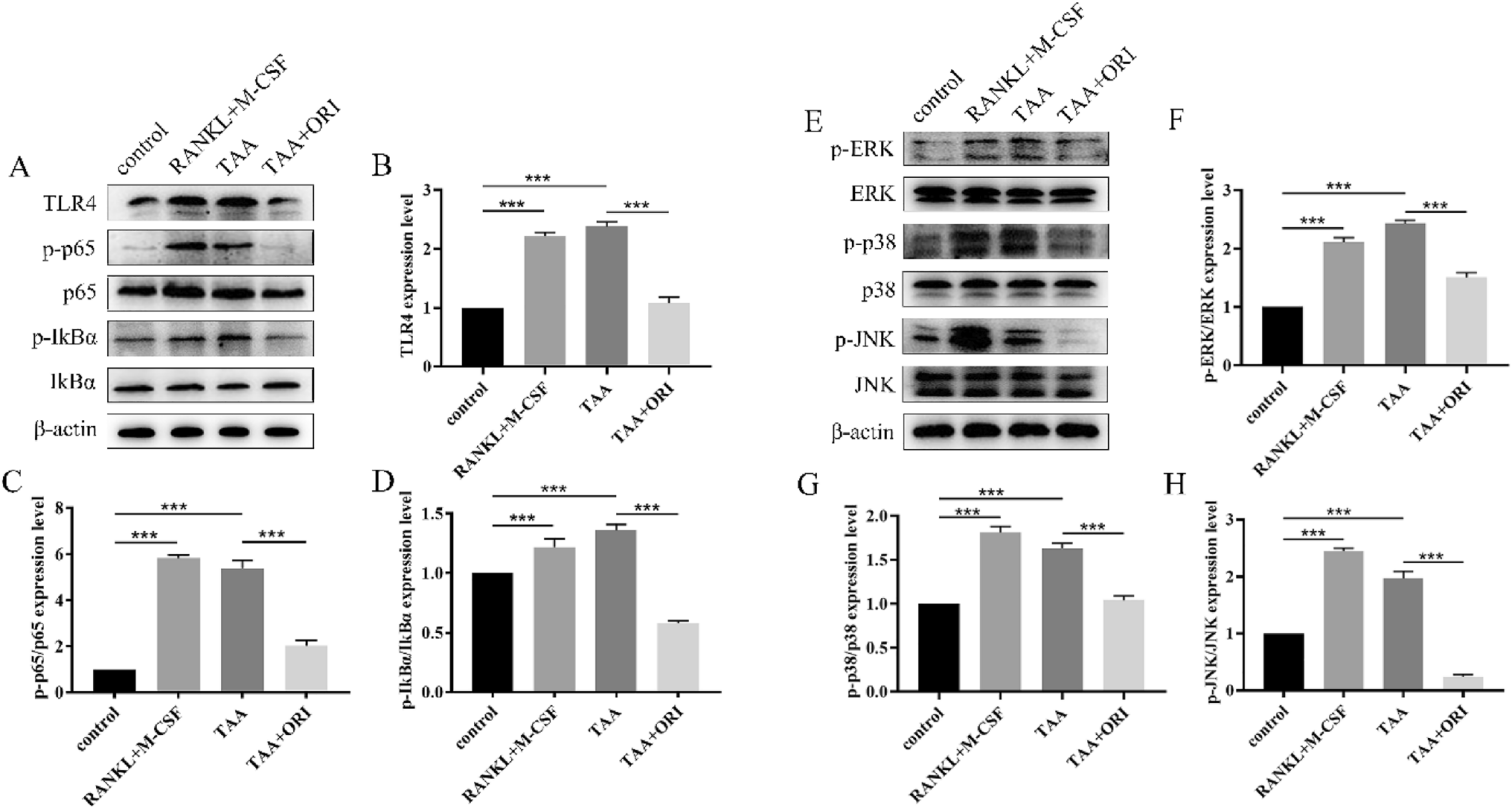
ORI inhibits TAA-induced activation of MAPK/NF-κB pathway. **A, E** Representative western blot images of the effects of TAA and ORI on MAPK/NF-κB pathway. **B–D, F–H** The band intensity ratios relative to TLR4(relatived to β-actin), p-p65, p-IκBα, p-ERK, p-p38 and p-JNK were quantified. Values were expressed as mean ± SD of three individual experiments; ***P < 0.001

Osteoclast differentiation is associated with the activation of MAPK/NF-κB pathways. Western blot showed that TAA could induce the phosphorylation of p65, IkBα, ERK, p38 and JNK to activate the MAPK/NF-κB pathway, while ORI can inhibit the phosphorylation of these proteins (Fig. 3).

**Fig. 4:**
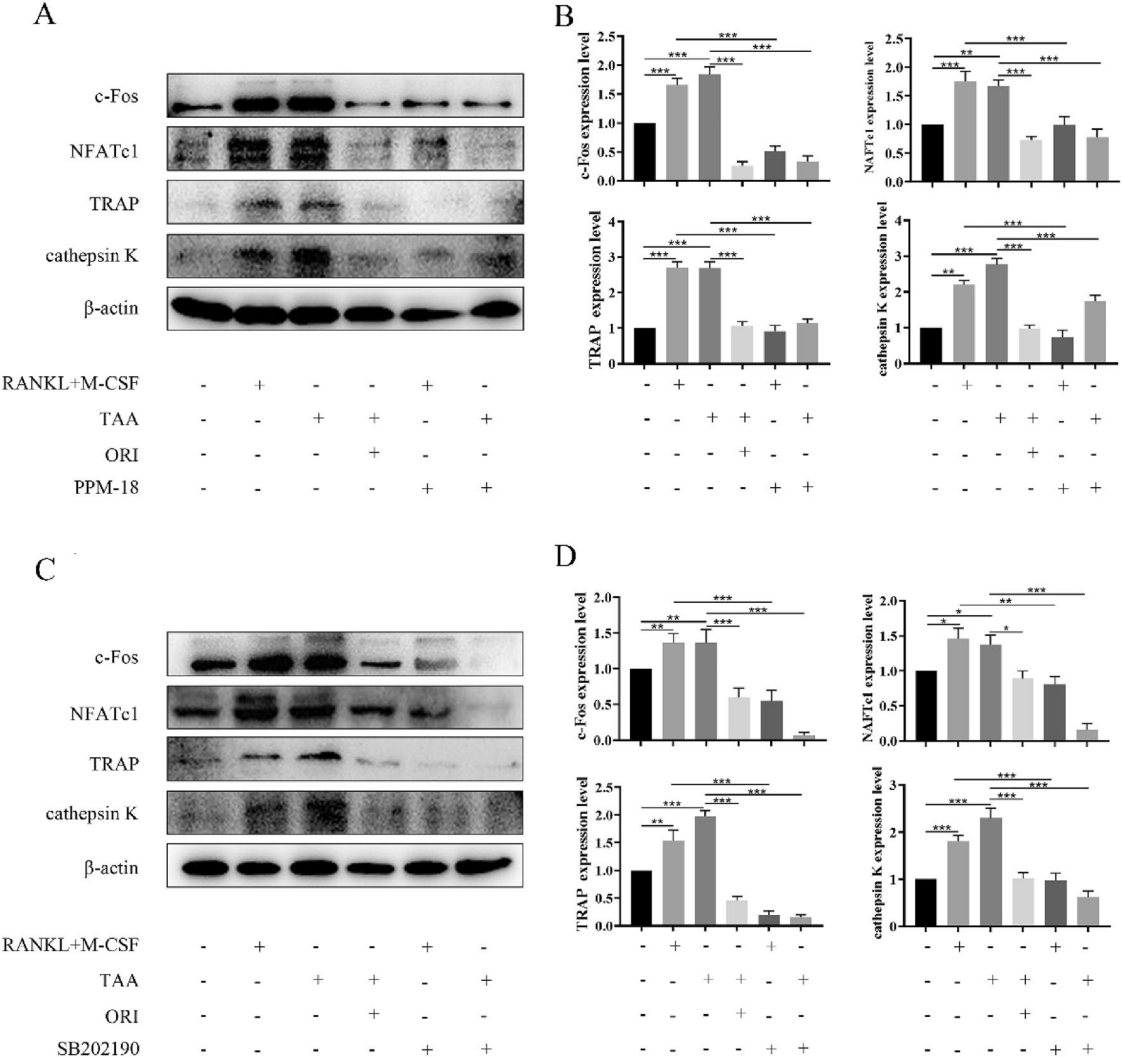
TAA promotes osteoclastogenesis by activating MAPK/NF-κB pathway, while ORI can inhibit this effect. **A, C** Osteoclast differentiation was performed after cells were treated with NF-κB inhibitor (PPM-18) or p38 MAPK inhibitor (SB202190) for 2 h. Representative western blot images of the effects of TAA and ORI on osteoclast differentiation. **B, D **The band intensity ratio of c-Fos, NFATc1, TRAP and cathepsin K relative to β-actin were quantified. Values were expressed as mean ± SD of three individual experiments; *P  <0.05, **P< 0.01, ***P < 0.001

To verify whether TAA indeed promotes osteoclast formation by promoting MAPK/NF-κB pathway, MAPK and NF-κB pathway inhibitors (p38 MAPK inhibitor, SB202190; NF-κB inhibitor, PPM-18) were used. The results showed that both MAPK and NF-κB pathways inhibitors could inhibit TAA-induced osteoclast-specific proteins expression, which was consistent with the effect of ORI (Fig. 4). These results suggested that TAA can promote osteoclastogensis by activating MAPK/NF-κB pathway, while ORI can inhibit osteoclast differentiation by inhibiting MAPK/NF-κB pathway.

### ORI Inhibits TAA-Induced p65 Nuclear Translocation and ROS Generation

**Fig. 5:**
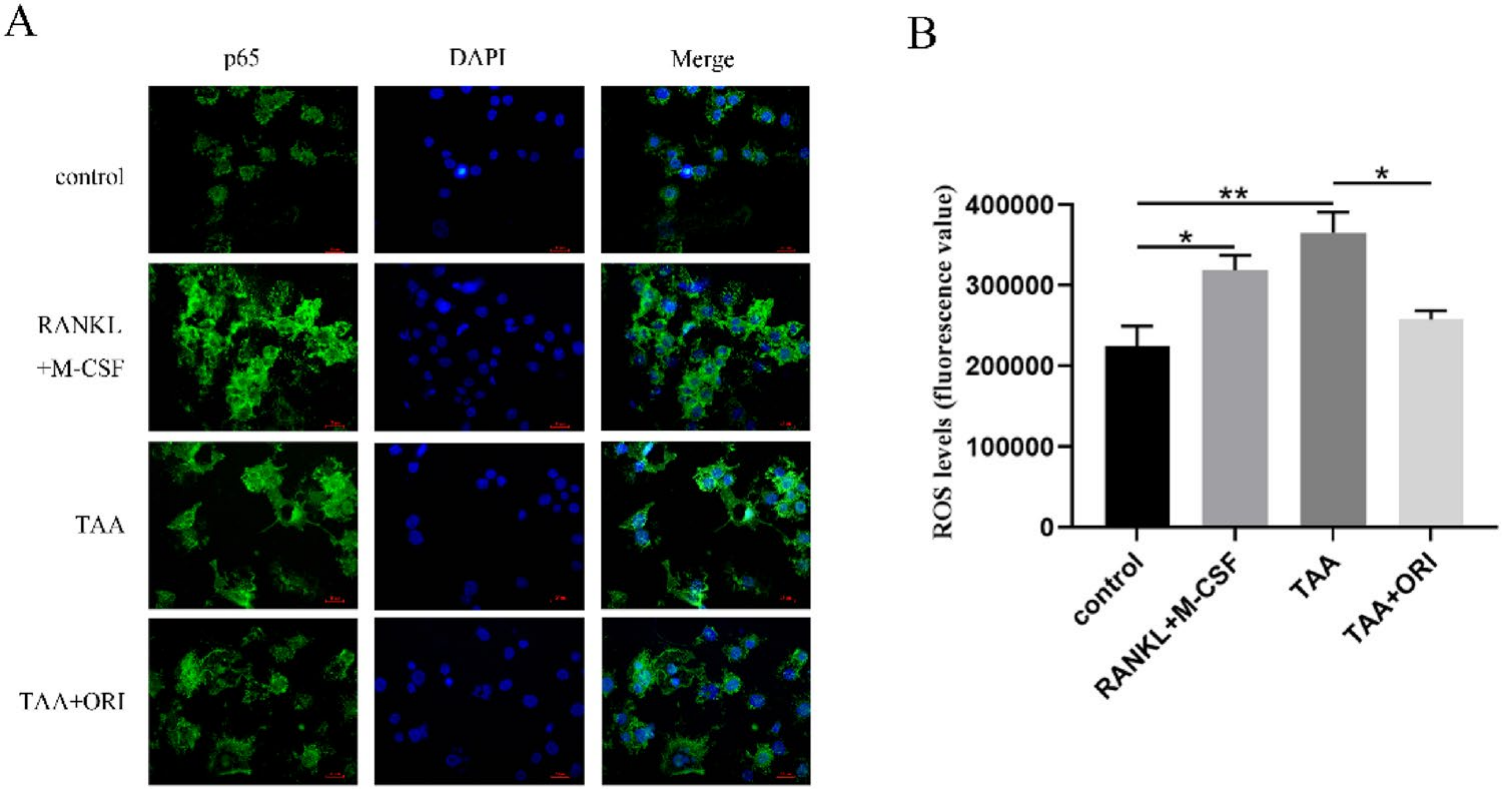
ORI inhibits TAA-induced p65 nuclear translocation and ROS generation. **A **After 7 days of osteoclast-induced differentiation, nuclear translocation of p65 was visualized by immunofluorescence. **B** Cells were cultured with different medium (50 ng/mL RANKL and 30 ng/mL M-CSF; 0.4 mg/ml TAA; 0.4 mg/ml TAA and 3.38 μM ORI) for 2 h, then intracellular reactive oxygen species levels were detected by flow cytometry. Values were expressed as mean ± SD of three individual experiments; *P < 0.05, **P < 0.01

Previous studies have found that RANKL can not only activate the NF-κB pathway, but also promote p65 nuclear translocation to induce osteoclast differentiation. In order to further elucidate the mechanism of the effects of ORI and TAA on osteoclast differentiation, we analyzed whether ORI and TAA could affect p65 nuclear translocation by immunofluorescence. As expected, TAA was able to promote p65 nuclear translocation, which was the same as the effect of RANKL and M-CSF. However, this effect can be significantly inhibited by ORI (Fig. 5A). In addition to this, ORI could also attenuate TAA-induced intracellular ROS production (Fig. 5B, 29.45% decrease vs TAA group).

### ORI Attenuates the Inhibition of TAA on Osteogenic Differentiation of BMSCs

**Fig. 6:**
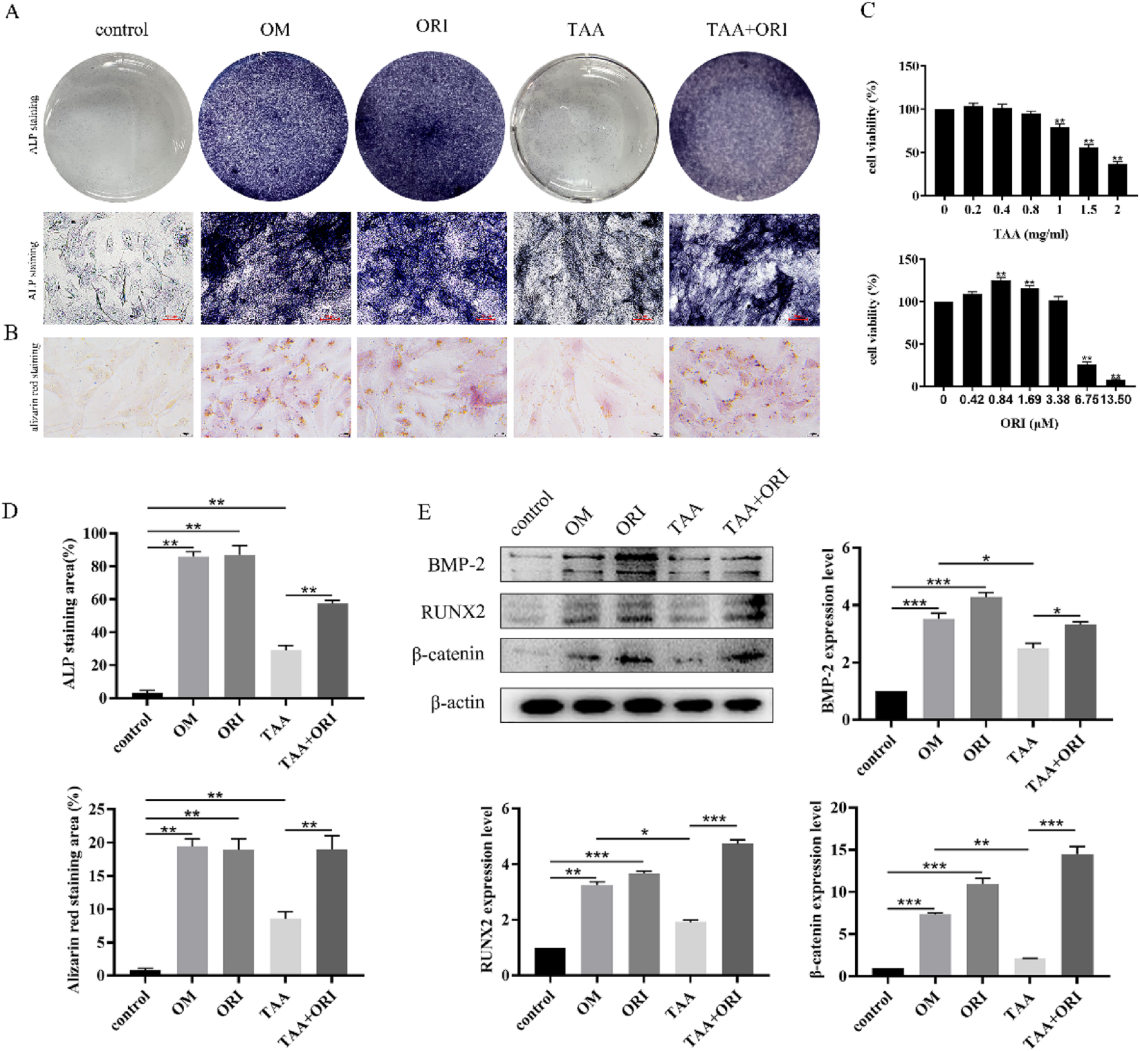
ORI attenuates the inhibition of TAA on osteogenic differentiation of BMSCs. **A** Expression of alkaline phosphatase in BMSCs cells treated with TAA or ORI for 7 days (OM: osteogenic medium). **B** Mineralized extracellular matrix was detected by Alizarin Red staining. **C **Viability of TAA-treated and ORI-treated BMSCs at 48 h. **D** The number of ALP-positive cells and the positive area of Alizarin Red staining were obtained after treatment with TAA or ORI. **E** After 7 days of osteogenic induction, the expressions of BMP-2, Runx2 and β-catenin were detected by western blotting. Results were normalized to the expression levels of β-actin. Values were expressed as mean ± SD of three individual experiments; *P < 0.05, **P < 0.01,  ***P < 0.001

BMSCs were used to explore the effects of TAA and ORI on osteogenesis. The results showed that TAA had no cytotoxicity to BMSCs at the investigated concentration (0 to 0.8 mg/ml). And ORI did not show significant cytotoxicity toward BMSCs at the investigated concentrations (0 to 3.38 μM) (Fig. 6C) Therefore, we selected 0.8 mg/ml TAA and 3.38 μM ORI as the subsequent experimental concentrations. Subsequently, ALP staining showed that TAA could inhibit ALP activity during osteogenic differentiation. The process of osteogenic differentiation is accompanied by the formation of mineralized bone nodules and the increased expression of osteogenesis-related protein including BMP-2, RUNX2 and β-catenin expression. As expected, TAA can inhibit the mineralization of BMSCs (Fig. 6B) and the expression of osteogenic specific genes (Fig. 6E). The inhibitory effect of TAA on osteoblast differentiation was significantly weakened after ORI treatment. More specifically, ORI could reverse the inhibitory effect of TAA on osteogenesis-related proteins (BMP-2, 32.80% increase vs TAA group; RUNX2, 147.64% increase vs TAA group; β-catenin, 583.96% increase vs TAA group).

### ORI Reverses TAA-Induced Apoptosis in BMSCs

**Fig. 7:**
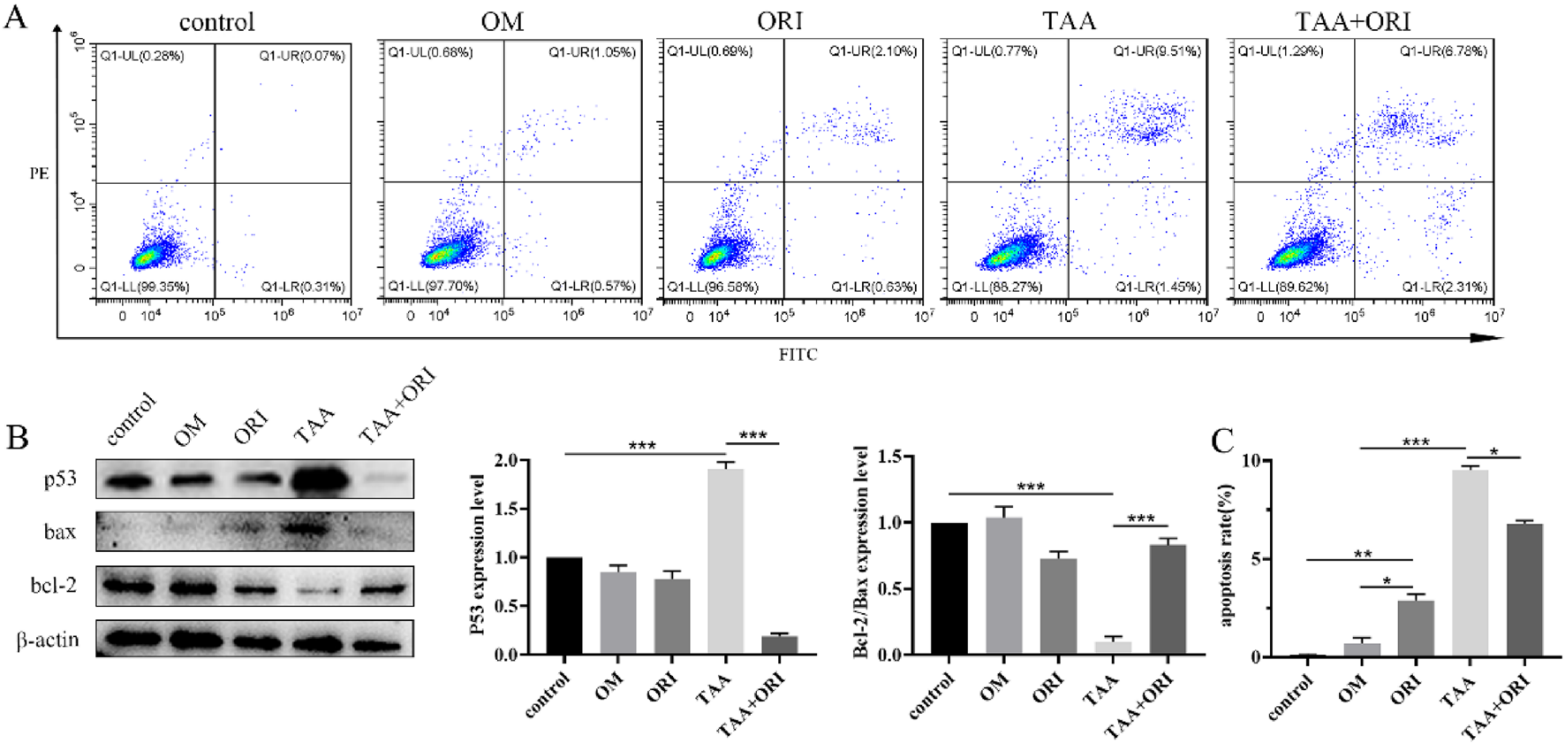
ORI reverses TAA-induced apoptosis. **A, C** The apoptosis rate of BMSCs cultured with TAA or ORI for 7 days was detected by flow cytometry. **B** The expression levels of the apoptosis-related proteins were detected by western blotting. Values were expressed as mean ± SD of three individual experiments; *P < 0.05, **P < 0.01, ***P < 0.001

In this study, TAA exposure could induce osteoblast apoptosis to a certain extent (Fig. 7A, C). It is obvious that the expression levels of apoptosis-related proteins bax and p53 increased significantly, while the expression level of bcl-2 decreased significantly. ORI can regulate apoptosis-related proteins to inhibit apoptosis (Fig. 7C, 28.71% decrease vs TAA group). This suggests that ORI may reverse the TAA-inhibited osteogenic differentiation by blocking apoptosis.

### ORI Inhibits TAA-Induced Adipogenic Differentiation of BMSCs

**Fig. 8:**
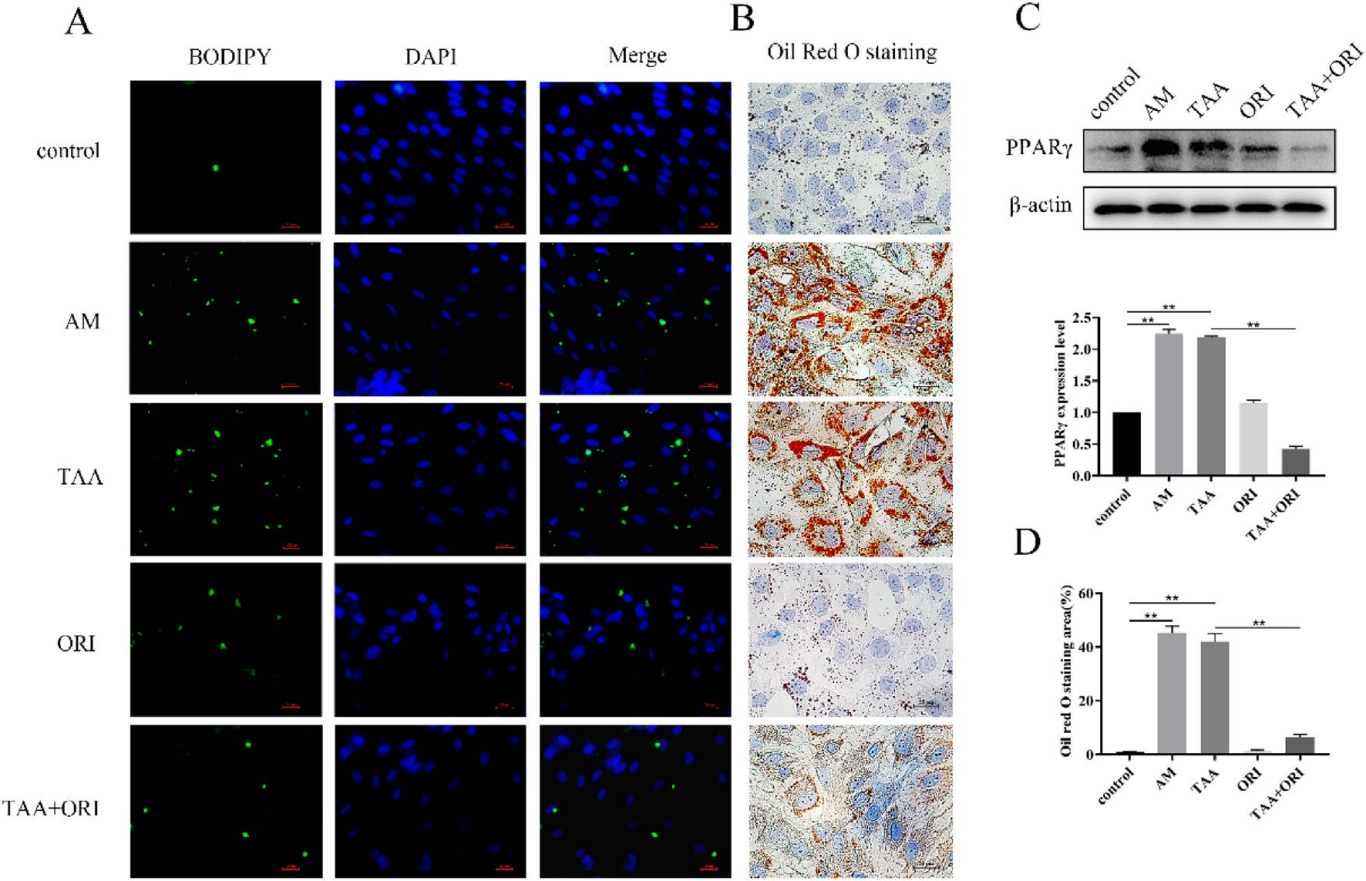
ORI inhibits TAA-induced adipogenic differentiation. **A, B, D** After 7 days of adipogenic induction, intracellular lipid accumulation was detected by BODIPY (**A**) and Oil Red O staining (**B**, **D**) (AM: adipogenic medium). **C** PPARγ expression levels of BMSCs was measured by western blotting. Values were expressed as mean ± SD of three individual experiments; *P < 0.05, **P < 0.01, ***P < 0.001

BMSCs can not only differentiate into osteoblasts, but also into adipocytes. After 21 days of adipogenic induction, BODIPY staining demonstrated that BMSCs had a large amount of lipid accumulation (Fig. 8A), and TAA can induce more and larger lipid droplets (Fig. 8B, D). ORI can prevent the formation of lipid droplets in BMSCs (Fig. 8D, 84.92% decrease vs TAA group) and inhibit the expression of the adipogenic-specific gene PPARγ (Fig. 8C, 80.46% decrease vs TAA group)), thereby inhibiting the adipogenic differentiation of BMSCs.

## Discussion

Increased bone resorption due to excessive osteoclast differentiation and reduced bone formation caused by blocked osteoblastic differentiation are manifestations of impaired bone homeostasis. The imbalance of bone homeostasis is the important contributors to osteoporosis [27, 28]. Many studies have shown that bone loss is related to inflammatory diseases, and some inflammatory drugs are associated with bone damage. At the same time, inflammation is associated with directed differentiation of stem cells and bone damage. In bone metabolism, various lytic bone diseases such as osteoporosis, osteoarthritis, periprosthetic infection and inflammatory aseptic loosening of orthopedic implants are often caused by excessive activation of osteoclasts [29]. Inflammatory osteolysis is mainly caused by excessive osteoclasts induced by microbial products and inflammatory cytokines [30]. Zhang found that wear particles could increase the number of osteoclasts and the area of osteolytic in mice, resulting in inflammatory osteolysis [31]. Lipopolysaccharide can also cause bone loss by inducing excessive inflammatory reactions and osteoclast activity, so it is commonly used to establish animal models of inflammatory osteolysis [32]. ORI is the main component of Rubescens chinensis in Lamiaceae. Although ORI has poor water solubility and low bioavailability, it is valuable for the broad-spectrum anti-cancer effects, effective anti-inflammatory, neuroprotective and bactericidal effects [33, 34]. However, the role of ORI in osteoporosis has rarely been reported. Here, we demonstrate that ORI can not only inhibit TAA- activated MAPK/NF-κB pathway to inhibit osteoclastogenesis, but also antagonize TAA to stimulate osteoblast differentiation, thereby regulating bone remodeling. The accumulation of intracellular ROS is an important factor leading to the formation of osteoclasts. Excessive ROS can activate IκBα phosphorylation, which in turn induces the translocation of p65 to the nucleus [35, 36]. In osteoclasts, p65 nuclear translocation and NFATc1 promoter binding can promote osteoclastogenesis, while ORI can inhibit the activation of this process by inhibiting p65 nuclear translocation [37, 38]. Our results showed that ORI could significantly inhibit TAA-induced IκBα phosphorylation, suggesting that ORI could block IκBα phosphorylation to inhibit the activation of NF-κB signaling pathway involved in osteoclast formation.

In addition to the NF-κB pathway, the activation of the MAPK pathway is also closely related to osteoclastogenesis [39]. The activation of the MAPK pathway leads to activation of the Jun protein, which is critical for osteoclast formation. Jun and c-Fos combine to form the complex AP-1, which can promote the expression of the nuclear transcription factor NFATc1 [40, 41]. Our results demonstrate that ORI can comprehensively inhibit the phosphorylation of ERK, JNK and p38, inhibiting the MAPK pathway to hinder osteoclastogenesis. The expression levels of osteoclast-specific proteins in the TAA + ORI -treated group were the same as those in the MAPK and NF-κB pathways inhibitor-treated groups. This suggested that ORI could inhibit TAA-induced osteoclastogenesis by inhibiting MAPK/NF-κB pathways.

We also found that ORI could not only inhibit TAA-induced osteoclast formation in vitro, but also antagonize TAA to promote osteogenic differentiation. As an early marker of osteogenesis, ALP could enhance mineralization during osteogenesis [42]. In this study, ALP staining showed that TAA could inhibit the activity of ALP, and alizarin red staining indicated that TAA could inhibit the formation of calcium nodules during the osteogenesis of BMSCs. ORI can antagonize TAA to promote the osteogenic differentiation of BMSCs. Wnt/β-catenin signaling pathway plays a significant role in determining the osteogenic differentiation of BMSCs. Zhang [43] found that Wnt signaling promoted BMP-2 expression upstream, and BMP-2 induced ALP expression was also regulated by the Wnt signaling pathway [44]. Interestingly, Chen found that BMP-2 was also able to regulate the expression of Wnt ligands to stimulate β-catenin-mediated signaling [45]. As a key transcription factor during osteoblast differentiation, increased activity of RUNX2 promoted osteoblast formation [46]. As shown by western blotting, we found that ORI could relieve the expression of key osteogenic proteins RUNX2, BMP-2 and β-catenin which were inhibited by TAA, and induce osteogenic differentiation of BMSCs. At the same time, we also found that TAA can affect the expression of apoptosis-related proteins and promote the apoptosis of BMSCs, while ORI can alleviate this effect.

BMSCs have the potential of multi-directional differentiation. It is well known that BMSCs can differentiate into adipocytes in addition to osteoblasts [25]. With the increase of age, BMSCs tend to differentiate into adipocytes rather than osteoblasts, resulting in the decrease of the number of osteoblasts [47]. Moreover, the number of adipocytes increases, eventually promoteing the formation of osteoporosis. Researches showed that PPARγ as a positive regulator of BMSC adipogenes, it played a key role in adipocyte formation [48, 49]. Our study found that ORI could inhibit TAA-induced adipogenic differentiation by attenuating the accumulation of lipid droplets in the cytoplasm and reducing the expression of PPARγ.

In conclusion, our research found that ORI can inhibit NF-κB/MAPK pathway and the generation of reactive oxygen species to alleviate TAA-induced osteoclast differentiation. At the same time ORI also has the function of promoting osteoblast differentiation and inhibiting adipogenic differentiation, which possibly through up- regulating BMP-2/RUNX2 pathway. This study shows that ORI, as a potential drug for the treatment of osteoporosis, has dual activities of inhibiting bone resorption and promoting bone formation.

## Data Availability

The data that support the findings of this study are available from the corresponding author upon reasonable request.

## Abbreviations

TAA: Thioacetamide
ORI: Oridonin
BMMs: Bone marrow macrophages
BMSCs: Bone mesenchymal stem cells
M-CSF: Macrophage colony-stimulating factor
RANKL: Receptor activator of nuclear factor kappa B ligand
OM: Osteogenic medium
AM: Adipogenic medium
